# Structural basis of RNA polymerase recycling by the Swi2/Snf2 family of ATPase RapA in *Escherichia coli*

**DOI:** 10.1016/j.jbc.2021.101404

**Published:** 2021-11-12

**Authors:** M. Zuhaib Qayyum, Vadim Molodtsov, Andrew Renda, Katsuhiko S. Murakami

**Affiliations:** Department of Biochemistry and Molecular Biology, The Center for RNA Molecular Biology, The Center for Structural Biology, Pennsylvania State University, University Park, Pennsylvania, USA

**Keywords:** RNAP recycling, RapA, post-termination complex, cryo-EM, AMPPNP, adenylylimidodiphosphate, CMPCPP, cytidine-5′-[(α,β)-methyleno]triphosphate, CTF, contrast transfer function, EC, elongation complex, NCI, National Cancer Institute, NTD, N-terminal domain, PDB, Protein Data Bank, PTC, post-termination complex, RNAP, RNA polymerase, ZBD, zinc-binding domain

## Abstract

After transcription termination, cellular RNA polymerases (RNAPs) are occasionally trapped on DNA, impounded in an undefined post-termination complex (PTC), limiting the free RNAP pool and subsequently leading to inefficient transcription. In *Escherichia coli*, a Swi2/Snf2 family of ATPase called RapA is known to be involved in countering such inefficiency through RNAP recycling; however, the precise mechanism of this recycling is unclear. To better understand its mechanism, here we determined the structures of two sets of *E. coli* RapA–RNAP complexes, along with the RNAP core enzyme and the elongation complex, using cryo-EM. These structures revealed the large conformational changes of RNAP and RapA upon their association that has been implicated in the hindrance of PTC formation. Our results along with DNA-binding assays reveal that although RapA binds RNAP away from the DNA-binding main channel, its binding can allosterically close the RNAP clamp, thereby preventing its nonspecific DNA binding and PTC formation. Taken together, we propose that RapA acts as a guardian of RNAP by which RapA prevents nonspecific DNA binding of RNAP without affecting the binding of promoter DNA recognition σ factor, thereby enhancing RNAP recycling.

Gene expression by RNA polymerase (RNAP) is a tightly regulated process throughout transcription cycle starting from promoter DNA binding, transcription initiation, elongation, and termination to RNAP recycling. Each step is controlled by interactions between RNAP core enzyme and regulatory factors. In bacteria, σ factors enable core RNAP to specifically recognize promoters and initiate transcription. RNA synthesis by RNAP triggers σ release from the core enzyme that allows multiple transcription factors binding to RNAP to modulate its function and activity until end of transcription cycle (1). General transcription elongation factors NusA and NusG bind RNAP to regulate the rate of transcription, half-life of transcription pausing, mediate transcription–translation coupling, and facilitate transcription termination (2, 3). The last phase of the transcription cycle, transcription termination, is controlled by ATP-dependent helicase/translocase Rho, which translocates along the nascent RNA and dislodges the elongation complex (EC) after interacting with RNAP, NusA, and NusG (4, 5) or, alternatively, by a termination RNA hairpin that interacts with NusA (6).

In addition to binding regulatory factors to RNAP, dynamics and flexibility of RNAP plays important roles in the transcription cycle. The structure of all cellular RNAPs resembles a crab claw, with the β and β′ subunits forming two “pincers” that surround the main channel of RNAP serving as the binding site for the DNA template (7). The pincer formed by the N-terminal and C-terminal regions of β′ subunit, also known as the “clamp,” has been shown to change its conformation during DNA binding and release. Single-molecule FRET studies as well as structural studies of bacterial RNAP revealed flexible nature of the clamp, which can acquire “open” and “closed” states (4, 8, 9, 10, 11). The clamp primarily remains in its “open” conformation in the RNAP core enzyme, but it closes upon formation of holoenzyme, open complex, and EC with DNA/RNA accommodated in the main channel of RNAP.

During the transcription termination, RNAP opens its clamp to release DNA template and RNA product to prepare for a next round of transcription cycle (RNAP recycling). However, some RNAPs form an undefined post-transcription/post-termination complex (PTC) (12) that prevents RNAP recycling, thus inhibiting gene expression. The PTC formation may be due to failure of DNA release from RNAP after ejecting RNA transcript or because of intrinsic affinity of RNAP core enzyme for DNA (nonspecific DNA binding) (13). DNA accommodated in the main channel of RNAP hinders the holoenzyme formation by disrupting an important interaction between β′ coiled-coil domain and σ domain 2. The PTC formation must be reduced to provide pool of free RNAP for gene expression.

Different bacterial species harbor distinct mechanisms of stimulating RNAP recycling. In firmicutes (*e.g.*, *Bacillus subtilis*) and actinobacteria (*e.g.*, *Mycobacterium smegmatis*), transcription factor HelD, a RNAP-associated *s*uper*f*amily 1 (SF1) ATPase, is involved in the RNAP recycling (14, 15). Recent cryo-EM studies of the RNAP–HelD complex (15, 16, 17) revealed the domain organization of HelD and its interactions with RNAP. These studies proposed a mechanism of RNAP recycling wherein HelD inserts two domains deep into the main and secondary channels of RNAP and promotes the clamp opening, destabilizing the EC and releasing DNA and RNA from RNAP. Dissociation of HelD from the RNAP is an active process that utilizes energy from ATP hydrolysis by HelD.

In proteobacteria including *Escherichia coli*, RNAP recycling is facilitated by RapA, a Swi/Snf2 protein superfamily ATPase (18) (Fig. 1*A*). RapA was identified about 50 years ago as a protein (named τ factor) copurifying with *E. coli* RNAP in a study that also discovered promoter specificity σ^70^ factor (19). The studies on Swi2/Snf2 family of enzymes have mostly focused on their roles in chromatin and nucleosome remodeling in eukaryotes (20). Interestingly, some members of the family, such as RapA, do not modify chromatin structure. Instead, its direct interaction with RNAP enhances RNA expression by facilitating RNAP recycling (21). Biochemical studies of RapA characterized its enzymatic activities and modes of binding to RNAP (22, 23, 24, 25, 26, 27), including (1) the ATPase activity of RapA stimulated by its association with RNAP, (2) RapA and σ^70^ competition for binding to RNAP, and (3) σ^70^ displacement of RapA from RNAP for holoenzyme formation. RapA stimulates transcription on supercoiled DNA, and its ATPase activity is essential for the RapA-dependent transcriptional activation. A model of RapA-mediated RNAP recycling proposes that RapA binds to PTC, remodels it by utilizing its ATPase activity, and releases DNA from RNAP (21). The X-ray crystal structure of RapA revealed the organization of its ATPase catalytic domains: two RecA-like lobe domains and two Swi/Snf2-like domains together forming the ATP-binding pocket (Fig. S1*A*) (28), which is distinct from the structure of HelD. The X-ray structure of the *E. coli* EC bound to RapA (EC–RapA) revealed that RapA binds around the RNA exit channel of RNAP but does not undergo conformational changes relative to the structure of apo RapA (29) (Fig. S1*D*). This study speculated that RapA reactivates a stalled EC by means of an ATP-driven backward translocation mechanism. However, a mechanism of RNAP recycling by RapA was not discussed.

**Figure 1:**
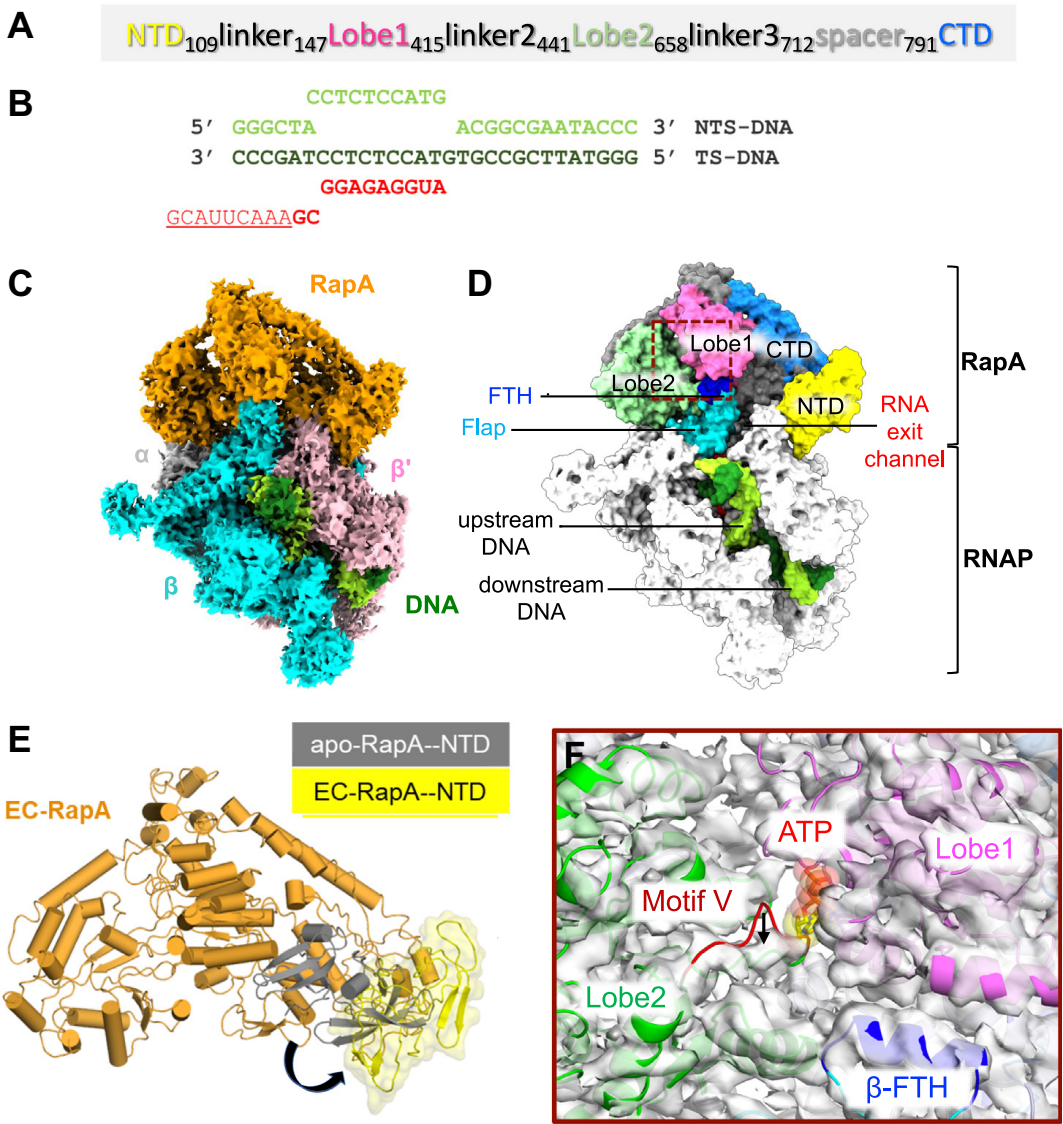
**Cryo-EM structure of the RNAP–RapA elongation complex.***A*, the domain organization of RapA. *B*, the sequence of the nucleic acid scaffold used to form the elongation complex. *C*, orthogonal view of the cryo-EM density map of EC–RapA. Subunits of RNAP and RapA are colored and labeled. Template and nontemplate DNA are shown in *dark green* and *light green*, respectively. *D*, organization of EC–RapA. The structure of EC–RapA is depicted as a surface model. RapA and RNAP domains are colored and labeled (FTH, flap-tip helix). The location of upstream and downstream DNA and the RNA exit channel are indicated. *E*, movement of RapA-NTD upon binding to RNAP. RapA is depicted as a cartoon model. RapA-NTD in the EC–RapA (this study) and in the apo-form RapA (Protein Data Bank: 6BOG) are colored *yellow* and *dark gray*, respectively. The shift of RapA-NTD is indicated by an *arrow*. *F*, allosteric opening of the ATP-binding site of RapA upon RNAP binding (*brown dashed box* in panel *D*). The apo-form RapA structure (*cartoon model*) fitted in the cryo-EM map of EC–RapA (*white transparent*). The lobe domains of RapA are colored and labeled, and the ATP binding is indicated by a modeled ATP. Compared with the apo-form RapA, motif V of the lobe 2 domain is shifted in the EC–RapA complex as indicated by an *arrow*. RNAP, RNA polymerase.

To understand the mechanism of RNAP recycling by RapA, we determined four sets of cryo-EM structures, including the RNAP EC, RNAP EC with RapA (EC–RapA), RNAP core enzyme (RNAP), and RNAP core enzyme with RapA (RNAP–RapA binary complex). The structures reveal the conformational changes induced in RNAP and RapA upon their association. Based on the structural findings and results of ATPase assay of RapA and DNA-binding assay of RNAP, we propose that RapA functions in RNAP recycling by acting as a guardian; RapA prevents nonspecific association of RNAP with DNA to maintain pool of free RNAP.

## Results and discussion

### Cryo-EM structure of the EC–RapA complex

EC–RapA was reconstituted *in vitro* by mixing RNAP with a DNA/RNA scaffold (Fig. 1*B*) to form the EC followed by adding RapA. The complex was crosslinked with bis(sulfosuccinimidyl)suberate (BS3) and purified by gel filtration column. Cryo-EM data were collected after vitrifying the complex supplemented with adenylylimidodiphosphate (AMPPNP) (a nonhydrolyzable ATP analog) aiming to capture ATP-bound state of RapA and cytidine-5′-[(α,β)-methyleno]triphosphate (CMPCPP) (a nonhydrolyzable analog of CTP) to prevent RNAP backtracking. Unsupervised 3D classification of the particles in the processing steps revealed two distinct classes (Fig. S2), including EC–RapA (class 2; 10%) and EC (class 4; 36%). Bayesian polishing of particles gave final reconstructions of EC–RapA and EC at resolutions of 3.3 and 3.15 Å, respectively.

The EC–RapA structure shows well-defined cryo-EM densities for RNAP (except ω subunit), RapA, and the nucleic acid scaffold except nontemplate DNA strand in the transcription bubble, and single-stranded RNA (Fig. 1*C* and Movie S1). The density for CMPCPP binding at the *i* + 1 site of RNAP was observed (Fig. S3*A*), but AMPPNP was not found in the RapA active site (Fig. S3*B*). RapA binds near the RNA exit channel of RNAP (Fig. 1*D*) as observed in the X-ray crystal structure of EC–RapA (Fig. S1*D*) (29). Nonetheless, the cryo-EM structure of EC–RapA revealed the RNAP-induced conformational changes of RapA including rotation of N-terminal domain (NTD; residues 1–109) and opening the ATP-binding site compared with its apo-form RapA structure (28) (Fig. 1, *E* and *F*). The RapA-NTD rotates 90° and swings away from the lobe 1 domain (Fig. 1*E*). The NTD rotation, together with the lobe 1 domain creates a cavity that harbors the flap-tip helix (β subunit: 897–905) and the zinc-binding domain (ZBD; β′ subunit: 70–88) of RNAP. The RapA-NTD rotation widens the RapA active site allosterically; motif V of the lobe 2 domain moves down toward a lobe 2 helix (residues 540–553) (Fig. 1*F*). The spatial rearrangement of motif V, in turn, may facilitate diffusion of ATP into the active site of RapA, explaining enhancement of the ATPase activity of RapA upon binding to RNAP and EC (Fig. S4).

The cryo-EM structure of EC–RapA reveals that RapA-NTD moves away from the lobe 1 domain to widen the ATP-binding site of RapA. This suggests a mechanism by which the ATPase activity of RapA is stimulated upon its binding to RNAP and is consistent with a potential autoinhibitory function of RapA-NTD: prior to binding RNAP, the RapA-NTD resides close to the catalytic lobe domains and may prevent nonspecific ATP binding/hydrolysis to prevent unnecessary ATP consumption. Consistently, the deletion of RapA-NTD increases ATPase activity fivefold (30).

### RapA constraints nonspecific association of RNAP with DNA

HelD in *B. subtilis* and *M. smegmatis* is the functional counterpart of RapA in *E. coli*; these ATP-dependent motor enzymes bind RNAP and facilitate RNAP recycling. Recent structural studies of the RNAP–HelD complex revealed that HelD associates with the main and the secondary channels of RNAP and opens the RNAP clamp (Fig. S5*A*) to actively dissociate DNA/RNA from the main channel of RNAP (16, 17, 31). In sharp contrast, RapA accesses neither the main nor the secondary channels of RNAP but associates around the RNA exit channel of RNAP, and the RapA binding does not open the RNAP clamp (Figs. 1 and S5*B*), suggesting that it may exploit an alternative mechanism to prevent the PTC formation and facilitates RNAP recycling.

**Figure 2:**
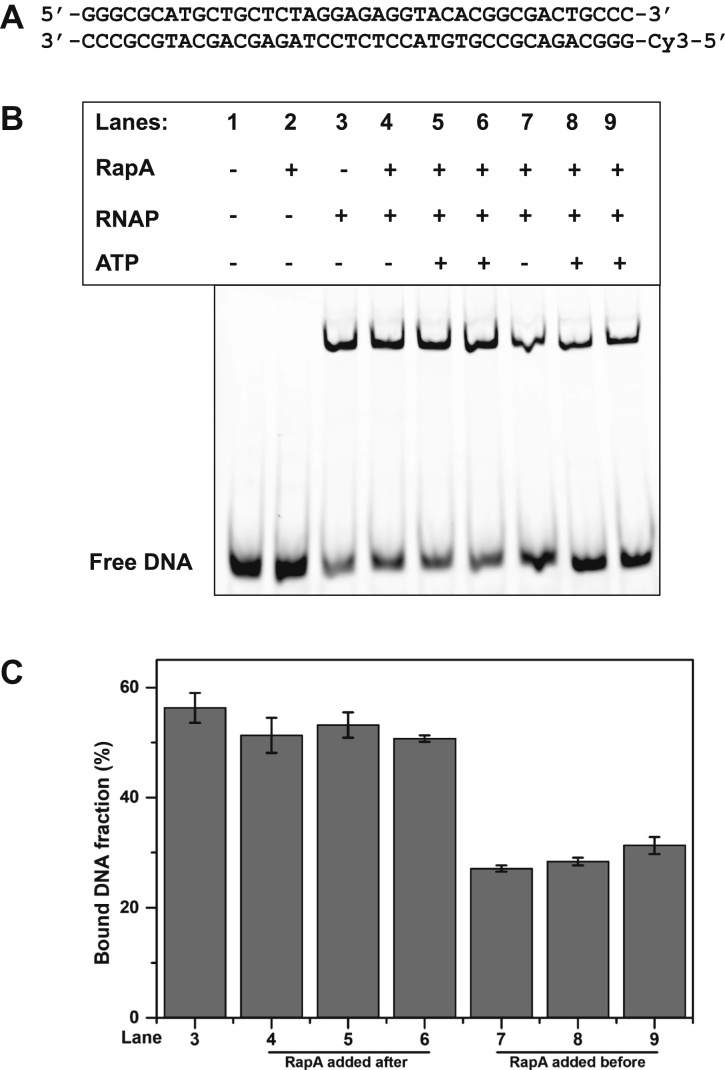
**RapA reduces nonspecific DNA binding of RNAP core enzyme.** EMSA to test the nonspecific DNA binding to RNAP in the absence and presence of RapA. *A,* the sequence of Cy3-DNA used in the assay. *B,* RNAP was mixed with Cy3-labeled DNA, and the shifted DNA bands were quantitated. + indicate the components in the reaction mixture. In lanes 4 to 6, RapA was added to preformed RNAP–DNA complex. In lanes 7 to 9, DNA was added to preformed RNAP–RapA complex. In lanes 5 and 8, 1 mM ATP was added together with RapA. In lanes 6 and 9, RapA was preincubated with 1 mM ATP for 10 min before addition. *C*, bar diagram showing the fraction of bound DNA (%) calculated with SE, n = 4. RNAP, RNA polymerase.

Nonspecific association of RNAP core enzyme with DNA (13) hampers RNAP recycling, reduces pool of free RNAP, and delays a next round of transcription. We, therefore, hypothesized that RapA may function as a guardian of RNAP core enzyme, by reducing its nonspecific binding of RNAP to DNA and preventing the PTC formation. To test this hypothesis, we performed EMSA using a fluorophore (Cy3)-labeled DNA (Fig. 2*A*) and quantitated the fraction of DNA bound to RNAP. About 60% of DNA was associated with RNAP in the absence of RapA (Fig. 2, *B* and *C*). Addition of RapA to RNAP reduced the formation of RNAP–DNA complex by ∼2.5 fold. Supplementing ATP to RapA had no effect on the RNAP and DNA association. RapA did not reduce the RNAP–DNA complex if it was added to the preformed RNAP–DNA complex even in the presence of ATP. These results support our hypothesis that RapA reduces nonspecific association of RNAP with DNA.

### Allosteric closure of RNAP clamp by RapA-NTD interaction with the zinc-binding domain of RNAP

**Figure 3:**
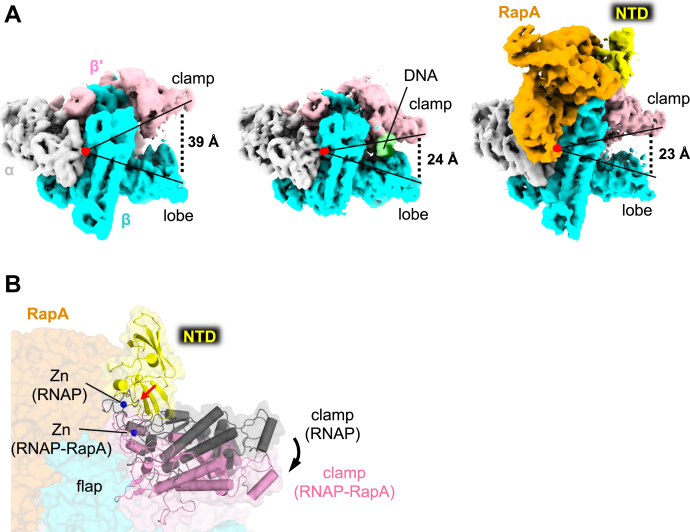
**Cryo-EM structures of the RNAP core enzyme and the RNAP–RapA binary complex.***A*, cryo-EM density maps of RNAP core enzyme (*left*), EC (*middle*), and RNAP–RapA (*right*). RNAP subunits and RapA are colored and labeled. RNAP domains and DNA are indicated. Opened and closed states of the RNAP clamp are represented by *acute-angled lines*, and distances between the clamp and lobe domains of RNAP are indicated. *B*, RapA-mediated clamp closure. The RNAP–RapA is depicted as a transparent surface with cartoon models of the RapA-NTD and RNAP clamp as well as CPK representation of Zn bound at the ZBD. The clamp and Zn of apo-form RNAP are also depicted (*gray*). The structures are superimposed by aligning the DPBB (double-psi-β-barrel) domains of RNAP. Steric clash between the RapA-NTD and ZBD in the opened clamp is indicated by a *red arrow*, and the RapA-induced closing of the RNAP clamp is indicated by a *black arrow*. EC, elongation complex; RNAP, RNA polymerase; ZBD, zinc-binding domain.

Because of flexible nature of the clamp, the RNAP core enzyme is prone to binding nonspecific DNA (13). Although RapA binds around the RNA exit channel of RNAP, it may close the RNAP clamp allosterically and prevent its nonspecific DNA binding. To study how the RapA influences the RNAP clamp conformation, we determined the cryo-EM structures of the RNAP core enzyme (Figs. 3*A* and S6) and the RNAP–RapA complex (Figs. 3*A* and S7) at nominal resolutions of 3.41 and 4.8 Å, respectively. In the RNAP–RapA complex (Fig. 3*A*), the RapA binds around the secondary channel of RNAP, and the RapA-NTD changes its orientation by contacting with the ZBD of RNAP as observed in the cryo-EM structure of the RNAP-EC (Fig. 1).

In the structure of RNAP core enzyme, the main channel widely opens for separating the β′ clamp and β lobe 39 Å apart (Fig. 3*A* and Movie S2). In contrast, the main channel is closed with the β′ clamp and β lobe distance of 23 Å in the RNAP–RapA complex (Fig. 3*A* and Movie S2) as observed in the cryo-EM structures of EC (24 Å) and EC–RapA (24 Å) determined in this study. The clamp closure of the RNAP–RapA binary complex is due to the positioning of RapA-NTD beside the ZBD of the β′ subunit (Fig. 3*B*), which is a pivot point of the clamp. Physical contact between RapA-NTD and ZDB may restrict the flexibility of clamp to keep it in a closed state allosterically. The ZBD of the largest RNAP subunit is conserved in all cellular RNAPs from bacteria to humans and serves as a platform for recruiting diverse accessory factors. For example, ZBD interacts with (1) bacterial protein SuhB (32) or phage protein Q (33, 34) to promote the formation of anti-PTCs; (2) the lead ribosome to couple transcription to translation (35, 36); and (3) Rho during transcription termination (4). In this study, we showed that the ZBD also recruits RapA, changing its conformation to activate the ATPase activity (Fig. 1*E*).

### Model for RapA-mediated RNAP recycling

**Figure 4:**
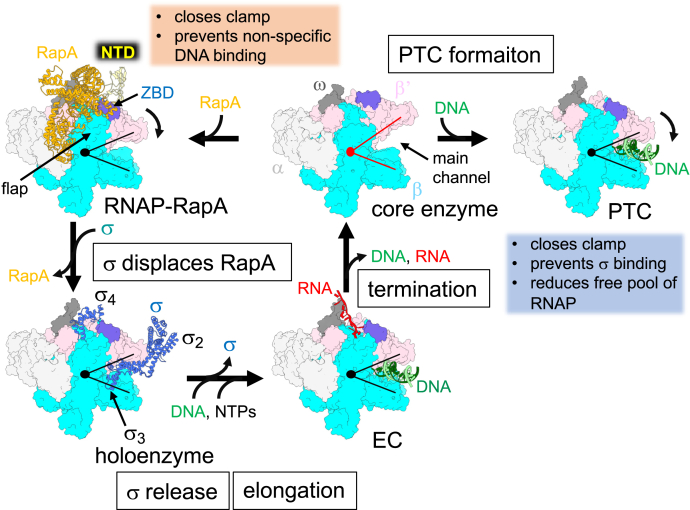
**RapA-mediated RNAP recycling.** Clamp dynamics of RNAP during transcription cycle (*left*) and PTC formation (*right*). RNAP core enzyme is shown as surface model, and opened and closed states of the RNAP clamp are represented by *acute-angled lines* (*red*, opened; *black*, closed). RapA, σ, DNA are depicted as ribbon models. Subunits, domains, and DNA binding main channel of RNAP are indicated. PTC, post-termination complex; RNAP, RNA polymerase.

The primary binding site of RapA on RNAP, β flap domain, is occupied by σ domain 4 when RNAP forms holoenzyme and by NusA NTD during transcription elongation (Fig. S8), indicating that RapA is unable to form a complex with RNAP during the initiation and elongation phases of transcription. We therefore propose that a stage of transcription cycle allowing RapA binding to RNAP, and influencing its activity is post-transcription termination when RNAP completes RNA synthesis and releases DNA template and RNA product (Fig. 4). We propose that RapA prevents the detrimental effects of RNAP bound at nonspecific DNA by acting as a recycling chaperone that binds RNAP core enzyme immediately after its release from DNA and RNA.

Nonspecific DNA binding in the main channel of RNAP not only prevents its promoter DNA binding but also hinders the σ factor binding on RNAP by blocking the interaction between σ domain 2 and the coiled-coil domain of β′ subunit. RapA captures RNAP core enzyme right after transcription termination and allosterically locks the RNAP in a closed clamp conformation to prevent its nonspecific association with DNA. RapA safeguards till it is replaced by σ factor to form holoenzyme (28).

In the RNAP and RapA complex, the β′ coiled-coil domain is available for recruiting the σ domain 2 (Fig. S8). We propose the mechanism of RapA displacement by σ as following: (1) the σ domain 2 binds the RNAP coiled-coil domain to form a ternary complex (RNAP–RapA–σ), (2) the σ domain 4 accesses the β flap domain to dissociate RapA from RNAP. The RapA displacement by σ may be enhanced by confirmational change of RapA upon the ATP binding and its hydrolysis at the active site of RapA.

Both RapA and HelD enhance transcription by facilitating the RNAP recycling using distinct mechanisms. RapA recycles in a passive manner by allosterically closing the clamp of RNAP core enzyme, whereas HelD participates by interacting with the main and secondary channels of EC to eject DNA/RNA from RNAP (Figs. 4 and S5). From bacterial genome sequence analysis, we found that the presence of RapA and HelD is mutually exclusive with the former being predominantly present in proteobacteria, whereas the latter is mostly present in fermicutes and actinobacteria. The existence of divergent recycling mechanisms reflects significant regulatory differences among bacteria that have been revealed by recent functional and structural analyses (2, 37, 38).

In summary, we propose that RapA functions as a guardian of RNAP after completion of transcription until the holoenzyme formation. RapA binding to RNAP core enzyme closes its clamp for reducing nonspecific DNA binding, thereby preventing PTC formation. Binding of σ factor to the RNAP–RapA binary complex competes out RapA (28), and the newly formed holoenzyme is ready to begin the next round of transcription cycle. Since RapA belongs to Swi/Snf2 superfamily ATPase, RapA may use its ATPase activity to stimulate its functions, including (1) closing the RNAP clamp and/or (2) dissociating RapA from core enzyme during σ binding. In addition, a recent single-molecule study on transcription termination revealed that after RNA transcript release from EC, majority of RNAP remain bound on DNA and exhibit one-dimensional sliding over thousands of base pairs (39, 40). HelD may remove DNA from such RNAP and DNA complex for RNAP recycling; however, it is not clear what role RapA would have in RNAP recycling. Further structural, biochemical, and biophysical studies of the RNAP and RapA interaction will be needed to complete our understanding of the transcription activation by RapA.

## Experimental procedures

### Protein purifications

*E. coli* RNAP core enzyme was overexpressed in *E. coli* BL21(DE3) cells transformed with pVS10 expression vector 2 (encoding α, β, C-terminally His_6_-tagged β′ and ω subunits) (41) and grown in LB medium supplemented with ampicillin (100 μg/ml) at 37 °C until an absorbance of ∼0.5 at 600 nm, and then IPTG was added (final concentration: 1 mM) and grown for 5 h. RNAP enzyme was purified using Polymin P precipitation followed by heparin (HiTrap Heparin column), Ni-affinity (HisTrap HP column), and anion exchange (MonoQ column) chromatography steps (all columns from GE Healthcare). The purified RNAP core enzyme (20 μM) was suspended in the storage buffer (10 mM Hepes, pH 7.5, 50 mM NaCl, 0.1 mM EDTA, pH 8.0, and 5 mM DTT), aliquoted, snap frozen in liquid nitrogen, and stored at −80 °C.

*E. coli* RapA protein was overexpressed in *E. coli* BL21(DE3) cells transformed with pQE80L expression vector (encoding N-terminally His_6_-tagged full-length RapA) and grown in LB medium supplemented with ampicillin at 37 °C till absorbance of 0.8 at 600 nm. Then the RapA expression was induced with IPTG (final concentration of 1 mM) at 37 °C for 3 h and harvested (28). The protein was purified by affinity and size-exclusion chromatography using prepacked 5 ml Ni-affinity (HisTrap HP column), 5 ml heparin (HiTrap Heparin column), and Superdex200 columns (all columns from GE Healthcare). The purified RapA (230 μM) in storage buffer (10 mM Hepes, pH 7.5, 50 mM NaCl, 0.1 mM EDTA, pH 8.0, and 5 mM DTT) was aliquoted, flash frozen in liquid nitrogen, and stored at −80 °C.

### Cryo-EM sample preparation

#### EC–RapA

The EC was reconstituted *in vitro* by mixing 4 μM RNAP core enzyme with equimolar amount of template DNA/RNA (Fig. 1*A*) in a storage buffer at 22 °C for 20 min, followed by mixing 6 μM nontemplate DNA for 10 min. The resulting EC was mixed with 5 μM RapA and incubated for 10 min at 22 °C. The EC–RapA complex was purified by a gel-filtration column (Superdex 200; GE Healthcare) to remove excess DNA/RNA and RapA. The EC–RapA was concentrated to 4 mg/ml using Amicon Ultra centrifugal filter with 5 kDa molecular weight cutoff (Merck Millipore). AMPPNP and CMPCPP (1 mM each) were added and incubated at 22 °C for 10 min, followed by BS3 (Sulfo DSS; 100 μM) crosslinking for 30 min at room temperature, and reaction was stopped by adding ammonium carbonate (1 M). The cross-linked EC–RapA was again passed through a gel-filtration column (Superdex 200) and concentrated to 2.5 mg/ml.

#### RNAP–RapA binary complex

The RNAP–RapA binary complex was assembled and purified in an identical fashion as the EC–RapA complex but omitting the nucleic acid scaffold and the nucleotide analogs. In brief, 4 μM RNAP was mixed with 5 μM RapA in a storage buffer and incubated for 10 min at 22 °C. The RNAP–RapA complex was passed through a gel-filtration column (Superdex 200) to remove excess RapA. The RNAP–RapA complex was concentrated to 2.5 mg/ml using Amicon Ultra centrifugal filter with 5 kDa molecular weight cutoff (Merck Millipore).

### Cryo-EM grids preparation

C-flat Cu grids (CF-1.2/1.3 400 mesh; Protochips) were glow-discharged for 40 s prior to the application of 3.5 μl of the sample (2.5–3.0 mg/ml protein concentration) and plunge-freezing in liquid ethane using a Vitrobot Mark IV (FEI) with 100% chamber humidity at 5 °C.

### Cryo-EM data acquisition and processing

#### EC–RapA

The grids were imaged using a 300 kV Titan Krios (Thermo Fisher Scientific) equipped with a K2-Summit direct electron detector at the National Cryo-EM Facility at the National Cancer Institute (NCI). A total of 1674 movies were recorded with Latitude software (Gatan, Inc) in counting mode with a pixel size of 1.32 Å and a defocus range of −1.0 to −2.5 μm. Data were collected with a dose of 4.64 e^−^/s/physical pixel, with an exposure time of 15 s (40 total frames) to give a total dose of 40 electrons/Å^2^. Data were processed using RELION (MRC Laboratory of Molecular biology), version 3.0.8. Dose-fractionated subframes were aligned and summed using MotionCor2 (UCSF Software) (42). The contrast transfer function (CTF) was estimated for each summed image using Gctf (MRC Laboratory of Molecular biology) (43). From the summed images, about 1000 particles were manually picked and subjected to 2D classification in RELION (44). Projection averages of the most populated classes were used as templates for automated picking in RELION. Autopicked particles were manually inspected and then subjected to 2D classification in RELION specifying 100 classes. Poorly populated classes were removed, resulting in a total of 718,074 particles. 3D classification of the particles was done in RELION using a model of *E. coli* core RNAP–RapA (Protein Data Bank [PDB] ID: 4S20) low-pass filtered to 60 Å resolution using EMAN2 (45) as an initial 3D template. Among the 3D classes, the best-resolved classes (class 2: 69,457 particles and class 4: 256,565 particles) were 3D autorefined. Bayesian polishing and CTF refinement were performed on the particles, and reconstructed maps were postprocessed in RELION (Fig. S2).

#### Core RNAP

The grids were imaged using a 300 kV Titan Krios (Thermo Fisher Scientific) equipped with a K3 Camera (Gatan, Inc) at the NCI. A total of 3072 movies were recorded with Latitude software in counting mode with a pixel size of 1.08 Å and a defocus range of −1.0 to −2.5 μm. Data were collected with a dose of 18 e^−^/s/physical pixel, with and exposure time of 3 s (40 total frames) to give a total dose of 45 electrons/Å^2^. The data were processed using cryoSPARC (Structural Biotechnology Inc) (46). Dose-fractionated subframes were aligned and summed using Patch-Motion correction job, and the CTF was estimated for each summed image using Patch-CTF. A total of 766,796 particles were autopicked using Topaz picker (47) and then subjected to two rounds of 2D classification. Bad classes were removed, resulting in a total of 510,364 particles. *Ab initio* model was generated, and the particles were subjected to multiple rounds of heterogeneous refinement. A final set of 235,617 particles was refined, and the reconstructed map was sharpened (Fig. S6).

#### RNAP–RapA binary complex

The grids were imaged using a 300 keV Titan Krios (Thermo Fisher Scientific) equipped with a K3 Camera at the NCI. A total of 3504 movies were recorded with Latitude software in counting mode with a pixel size of 1.08 Å and a defocus range of −1.0 to −2.5 μm. Data were collected with a total dose of 45 electrons/Å^2^. Movies were motion corrected using multipatch motion correction, and CTF values were estimated using multipatch CTF estimation in cryoSPARC (46). A total of 1,360,137 particles were autopicked using Topaz picker (47) and subjected to 2D classification job. Particles from selected good classes were used to generate initial models. All the particles were then further classified multiple times using heterogeneous refinement job type to discard bad particles. A final set of 102,128 particles were then imported to RELION. To improve the density near the β lobe, focused classification was performed on RNAP density. The good class containing 88,511 particles was selected, and the particles were refined and postprocessed (Fig. S7).

### Model building and refinement

To refine the EC–RapA complex structure, the crystal structure of EC–RapA (PDB ID: 4S20) was manually fit into the cryo-EM density map using Chimera (UCSF Resource for Biocomputing, Visualization, and Informatics) (48), DNA and RNA were manually built by using Coot (MRC Laboratory of Molecular biology) (49), and the initial model was real-space refined using Phenix (50). In the real-space refinement, the domains of RNAP and RapA were rigid-body refined and then subsequently refined with secondary structure, Ramachandran, rotamer, and reference model restraints. The cryo-EM structures of the RNAP–RapA binary complex, RNAP core enzyme, and RNAP–DNA–RapA complex were built using the cryo-EM structure of EC–RapA as a reference model, and these structures were refined as described in the EC–RapA complex structure refinement (Table S1).

### EMSA

A linear Cy3-labeled dsDNA was generated by annealing the template and nontemplate strands by heating to 95 °C for 5 min and subsequent cooling to 10 °C at 1.5 °C/min (Fig. 3*A*). A final concentration of 150 nM Cy3-dsDNA was incubated with 300 nM RNAP for 10 min at 37 °C, followed by the addition of 300 nM RapA (either in the absence or in the presence of 1 mM ATP) and incubated for another 10 min (Fig. 3*B*, lanes 4–6). In the experimental set, first RNAP–RapA complex was formed by mixing 300 nM RNAP and 300 nM RapA (with/without 1 mM ATP), followed by incubation for 10 min at 37 °C. Then, 150 nM Cy3-dsDNA was added to the preformed complex and further incubated for 10 min at 37 °C. Samples were loaded on a nondenaturing 4% polyacrylamide gel and electrophoresed in 0.5× Tris/borate/EDTA buffer. The Cy3-labeled DNA bands were visualized by a Typhoon imager and quantified using ImageQuant software (GE Healthcare).

### ATPase assay

RapA or RNAP–RapA or EC–RapA (150 nM) were mixed with 100 μl of reaction buffer (40 mM Tris–HCl, pH 7.7, 50 mM KCl, 10 mM MgCl_2_, and 1 mM ATP). After incubation for 10 min at 37 °C, the release of pyrophosphate by ATP hydrolysis was detected by the addition of 800 μl of color development solution containing 4.2% ammonium molybdate, 0.045% malachite green, and Tween-20, which was premixed and filtered through Whatman No. 1 filter paper. Color development was quenched after 1 min by adding 100 μl of 34% citric acid. After incubating for 30 min at room temperature, the absorbance at 660 nm was measured (51).

## Data availability

The cryo-EM density maps have been deposited in Electron Microscopy Data Bank under accession codes: EMD-23900 (EC–RapA), EMD-23901 (EC), EMD-23902 (core RNAP), and EMD-23903 (RNAP–RapA). Atomic coordinates for the reported cryo-EM structures have been deposited with the PDB under accession numbers 7KMN, 7KMO, 7KMP, and 7KMQ.

## Supporting information

This article contains supporting information.

## Conflict of interest

The authors declare that they have no conflicts of interest with the contents of this article.
