# Supplementary material for: Structural basis of RNA polymerase recycling by the Swi2/Snf2 family of ATPase RapA in Escherichia coli
Source: J Biol Chem. 2021 Nov 12;297(6):101404. doi: 10.1016/j.jbc.2021.101404 (PMC8666675; doi:10.1016/j.jbc.2021.101404)
Supplement: Figures S1–S8 and Table S1 [file mmc1.pdf]

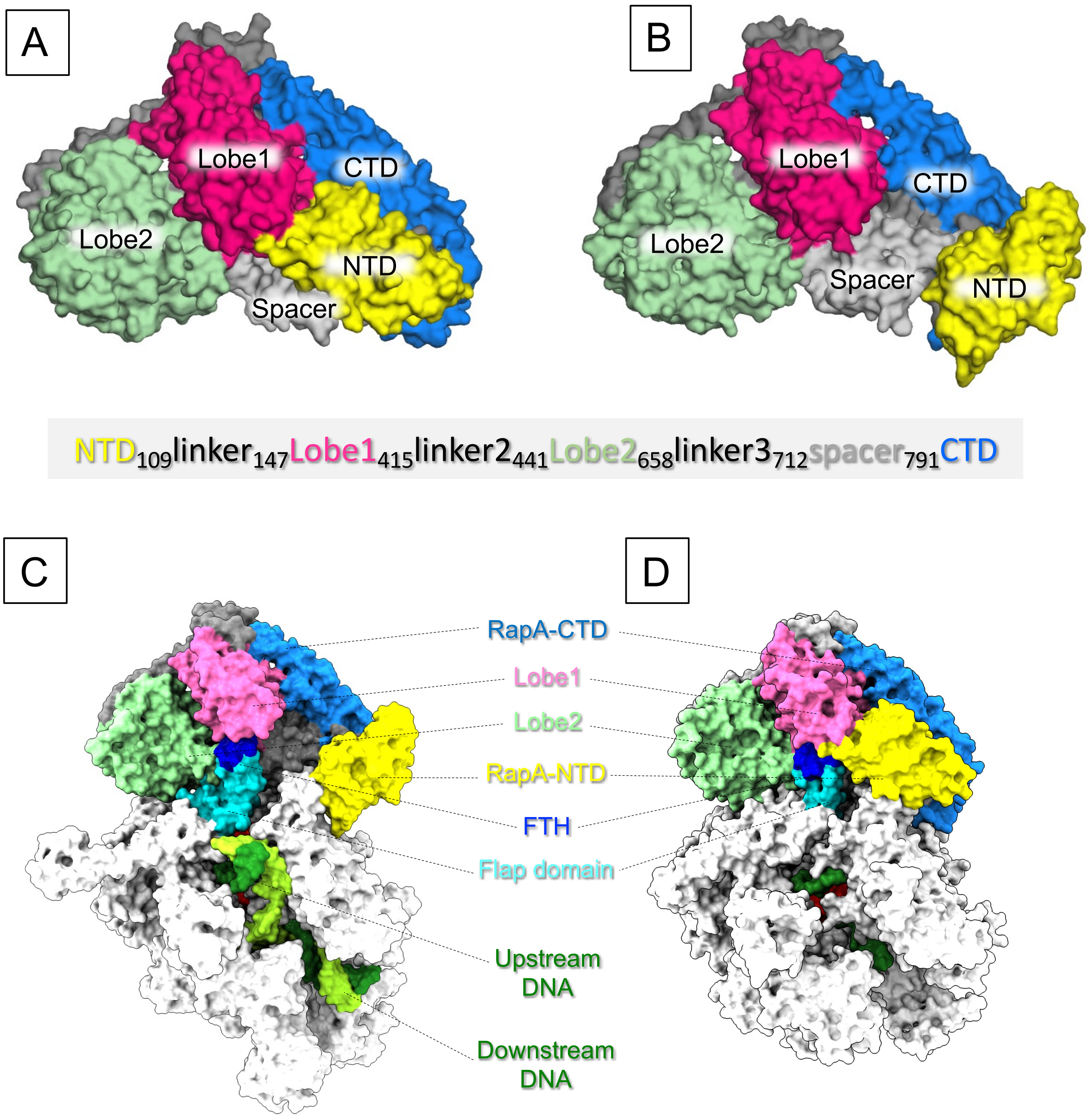

Cryo-EM structure of EC and RapA (this study) (PDB: 7MKN)

X-ray crystal structure of EC-RapA (Liu *et al.*, *PNAS* 2015) (PDB: 4S20).

SFigure 1. RapA conformation in apo-form and in RNAP bound form.

RapA conformations found in the crystal structure of apo-form RapA (**A**) and in the cryo-EM structure of EC-RapA (**B**). Comparison of the RapA conformations in the cryo-EM structure (**C**) and the X-ray crystal structure of EC-RapA (**D**) (28).

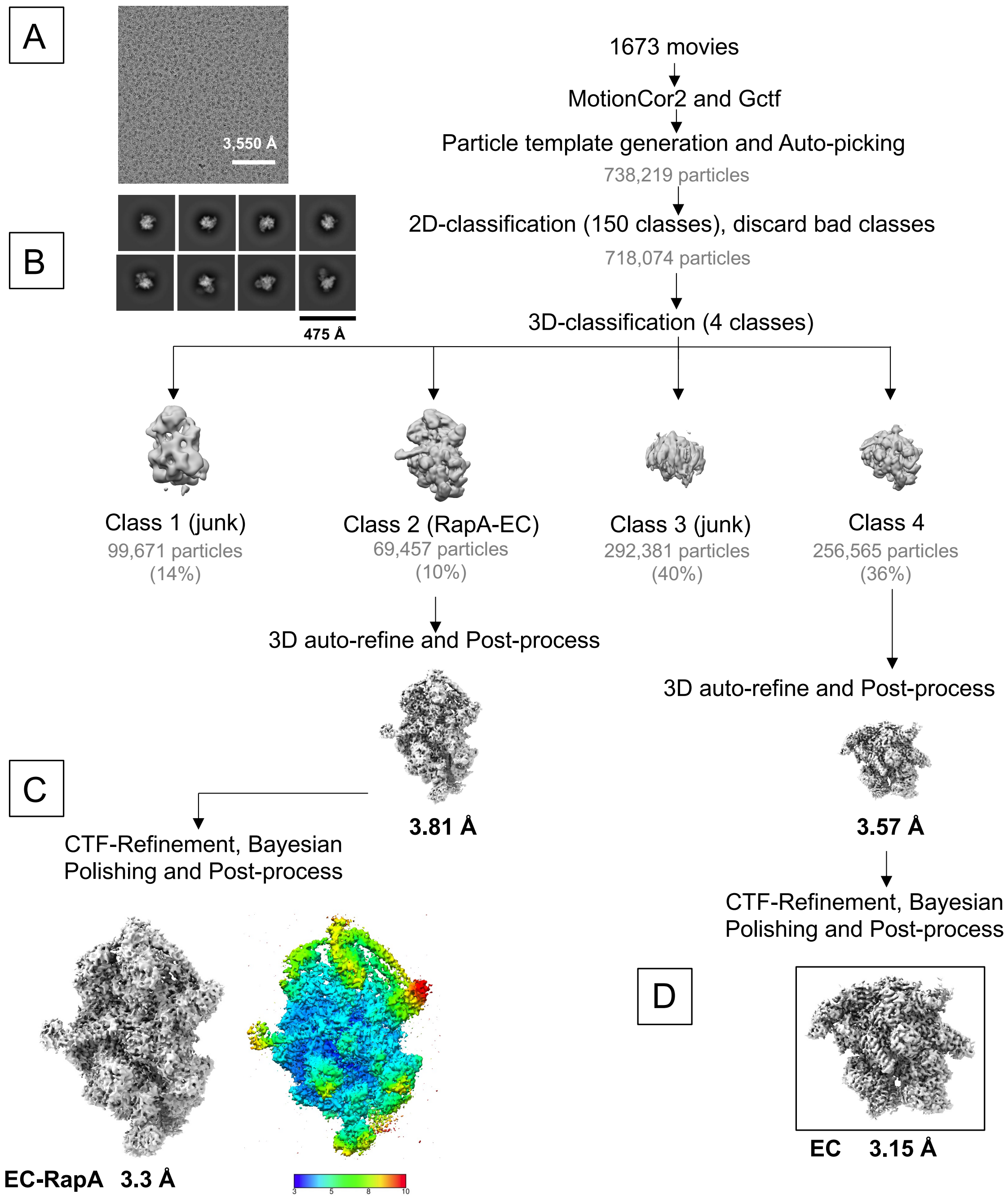

SFigure 2. Cryo-EM processing pipeline for RNAP-RapA EC.

A) A representative micrograph used for data processing.

B) Selected representative 2D classes from 2D classification (top, EC; bottom, EC-RapA).

C) The postprocessed cryo-EM density map of EC-RapA. The right view is identical to left but colored by local resolution.

D) The postprocessed cryo-EM density map of the EC.

A

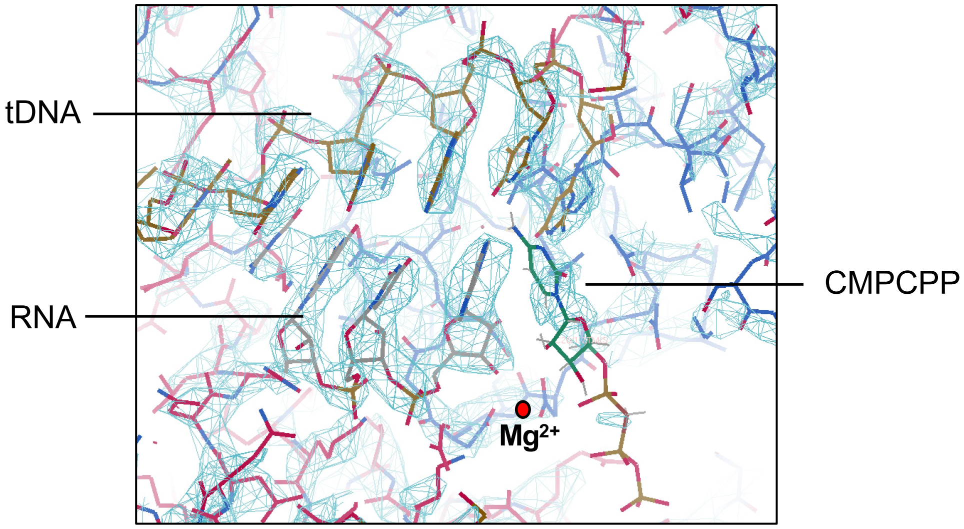

RNAP active site

B

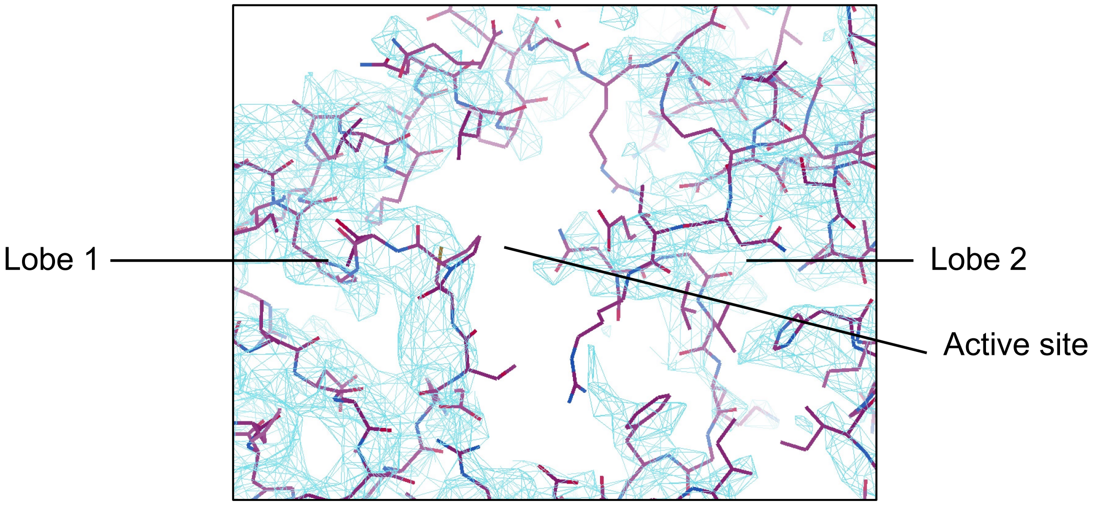

RapA active site

SFigure 3. Cryo-EM density maps (blue mesh) at the active sites of RNAP (A) and RapA (B) in the EC-RapA.

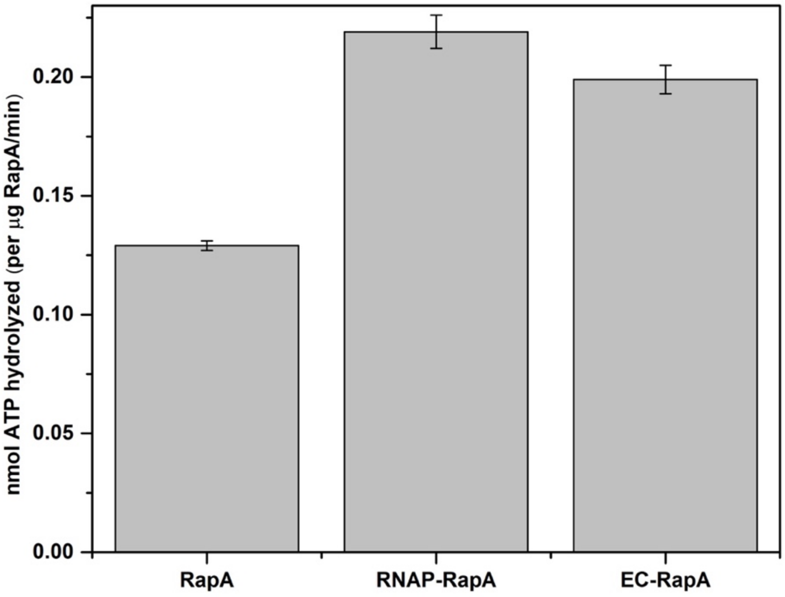

SFigure 4. ATPase activities of RapA as apo-form, in complex with RNAP and in complex with EC. RapA shows enhancement of its ATPase activity when bound to RNAP and EC. S.E. calculated with n=3.

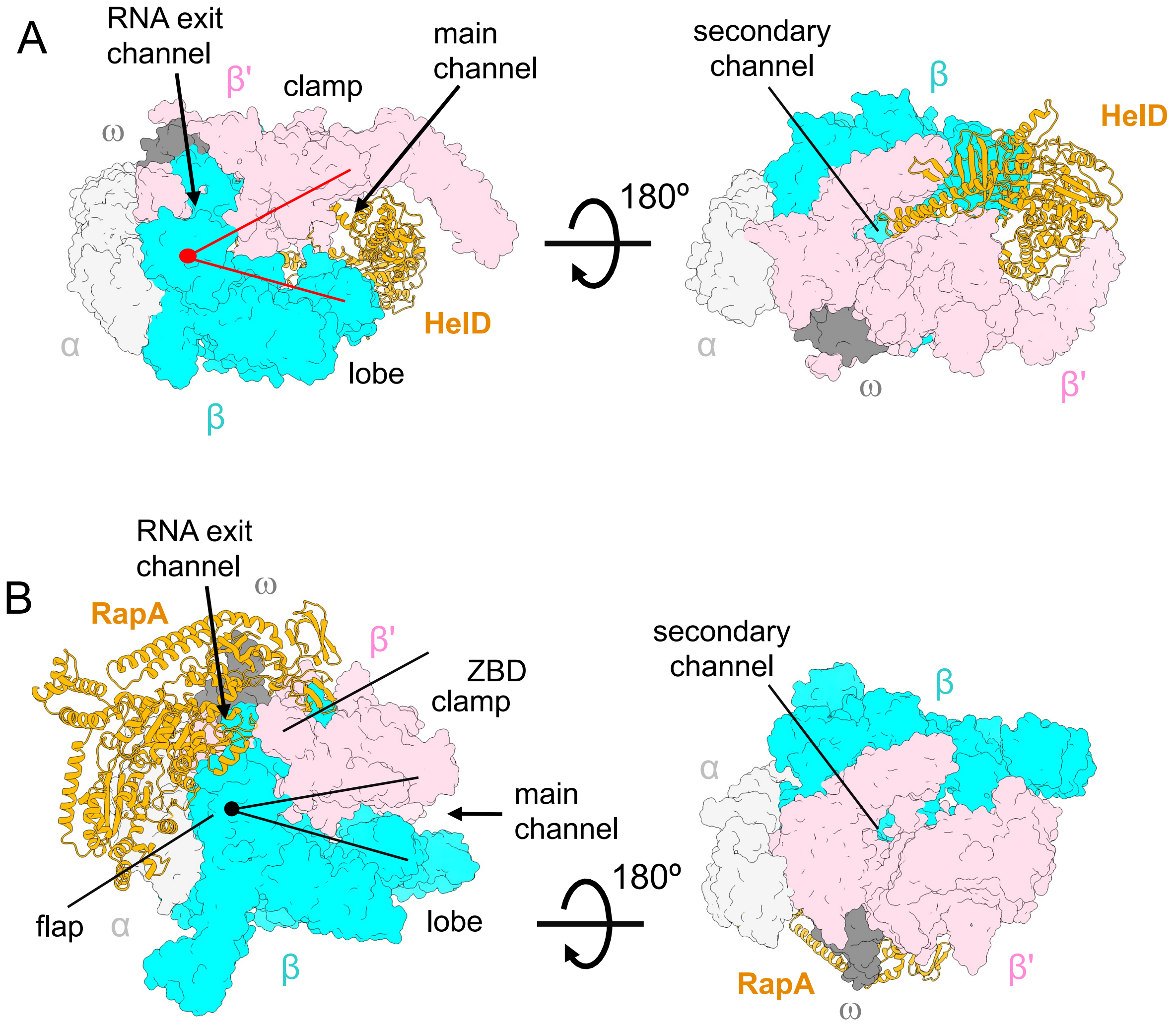

SFigure 5. A) Comparison of the structures of RNAP-HelD complexes (A) (PDB: 6YXU) and RNAP-RapA (B) (PDB: 7MKQ). The RNAP core enzymes (A: *E. coli*, B: *Mycobacterium smegmatis*) are shown as surface models and RapA and HelD are shown as ribbon models. Subunits, domains, and channels of RNAP are indicated. Opened (**A**) and closed (**B**) states of the RNAP clamp are represented by acute-angled lines (red, opened; black, closed).

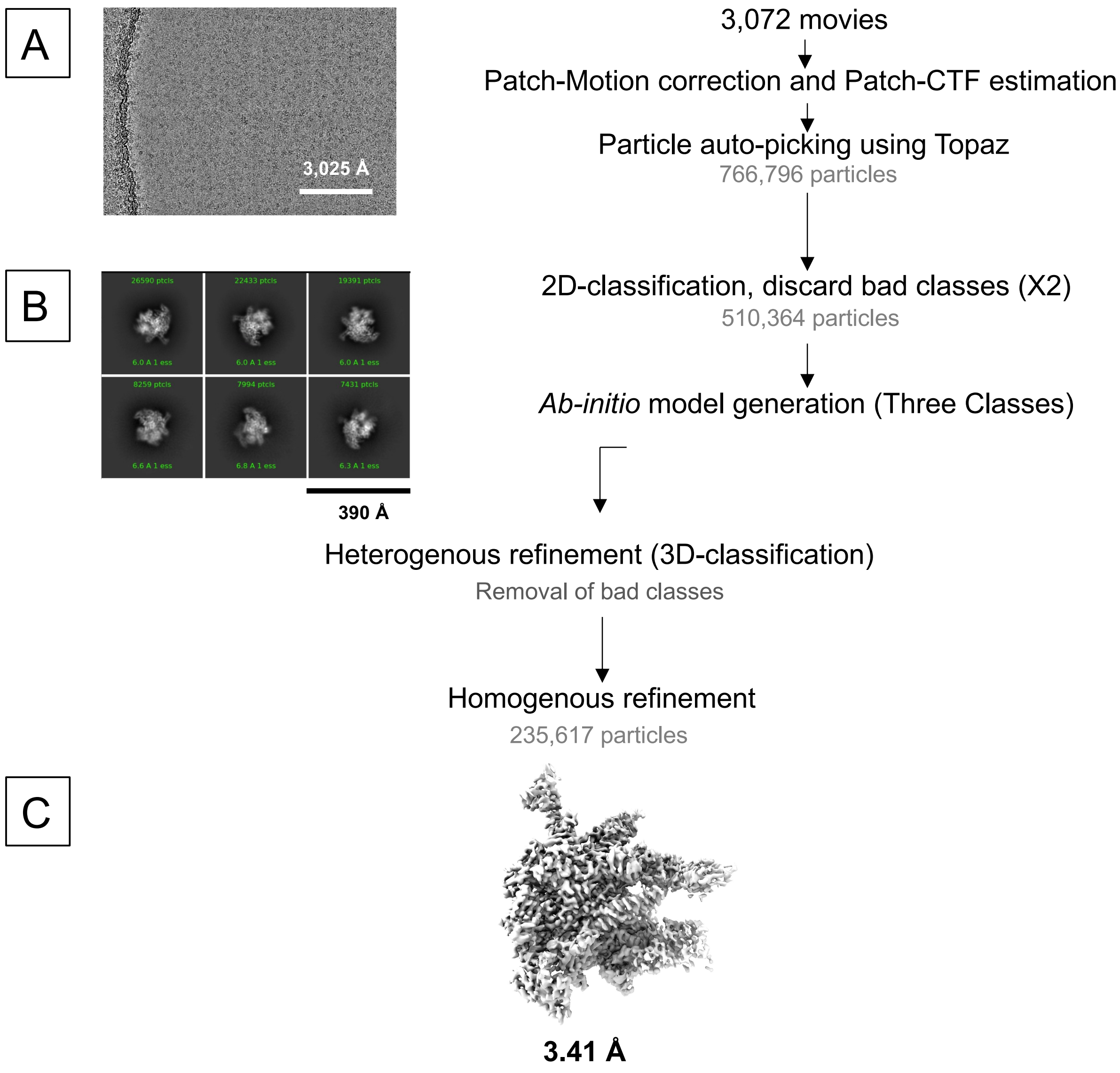

SFigure 6. Cryo-EM processing pipeline for RNAP core enzyme.

A) A representative micrograph used for data processing.

B) Selected representative 2D classes from 2D classification.

C) The postprocessed cryo-EM density map.

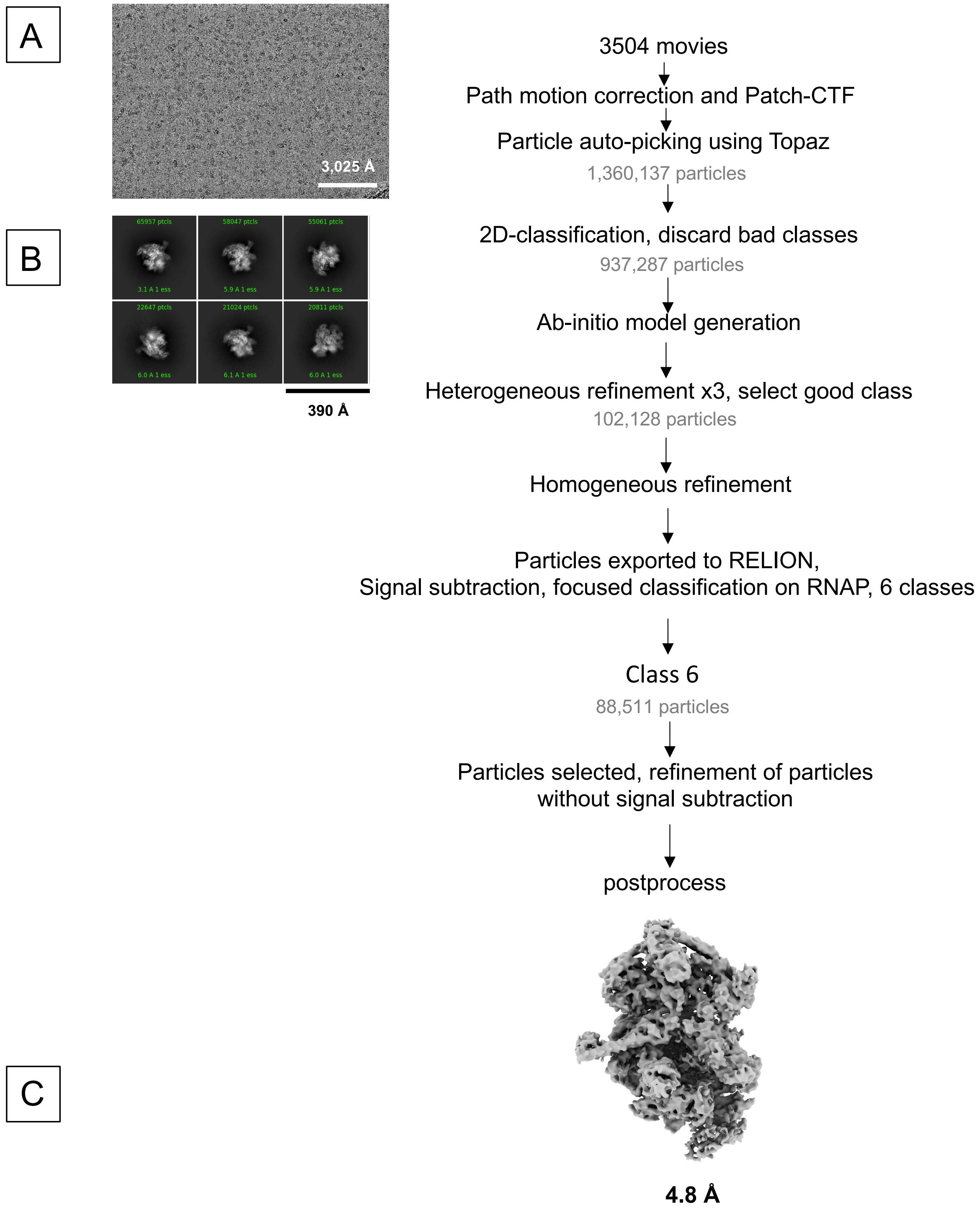

SFigure 7. Cryo-EM processing pipeline for RNAP-RapA binary complex.

A) A representative micrograph used for data processing.

B) Selected representative 2D classes from 2D classification.

C) The postprocessed cryo-EM density map.

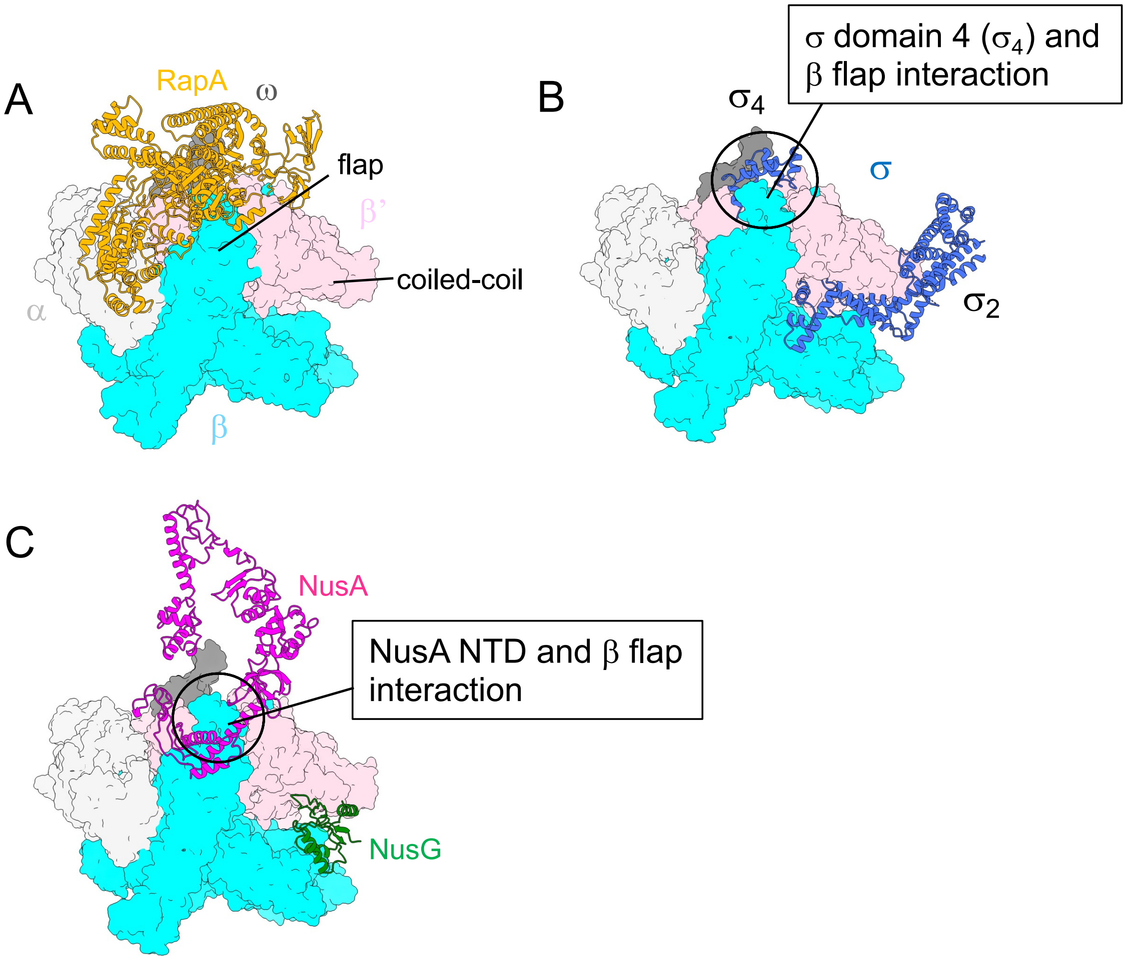

SFigure 8. Binding sites of σ and elongation factors NusA/NusG on RNAP compared to the RapA binding site. RNAP core enzyme is shown as surface model and RapA (A), σ^{70} (B) and NusA/NusG (C) are are depicted as ribbon models. Subunits and domains of RNAP are indicated. Binding sites of σ^{70} and NusA that overlapped with the RapA binding site are indicated by black circles.

SMovie 1: Cryo-EM structure od *E. coli* RNAP elongation complex with RapA

SMovie 2: RapA-mediated allosteric closure of the RNAP clamp

Supplementary Table 1. Cryo-EM data collection, refinement, and validation statistics

	EC-RapA (EMD-23900) (7MKN)	EC (EMD-23901) (7MKO)	coreRNAP (EMD-23902) (7MKP)	RNAP-RapA (EMD-23903) (7MKQ)
Data collection and processing				
Magnification	105,000	105,000	81,000	81,000
Voltage (kV)	300	300	300	300
Electron exposure (e^-/Å^2)	40	40	45	45
Defocus range (μm)	-1.0 to -2.5	-1.0 to -2.5	-1.0 to -2.5	-1.0 to -2.5
Pixel size (Å)	1.32	1.32	1.08	1.08
Symmetry imposed	C1	C1	C1	C1
Initial particle images (no.)	718,074	718,074	275,629	275,629
Final particle images (no.)	69,457	256,565	49,995	79,275
Map resolution (Å)<br>FSC threshold	3.3<br>0.143	3.15<br>0.143	3.41<br>0.143	4.8<br>0.143
Map resolution range (Å)	3.0-10.0	3.0-11.0	2.8-6.3	2.7-12.2
Refinement				
Initial model used (PDB code)	4S20	7MKN	7MKN	7MKN
Model resolution (Å)<br>FSC threshold	3.3<br>0.143	3.15<br>0.143	3.41<br>0.143	4.8<br>0.143
Model composition<br>Non-hydrogen atoms<br>Protein residues<br>Ligands	 34,084<br>4,177<br>Zn:2, Mg:1, 2TM	 26,269<br>3,194<br>Zn:2, Mg:1 2TM	 24,943<br>2,986<br>Zn:2, Mg:1	 32,822<br>4,182<br>Zn:2, Mg:1
B factors (Å^2)<br>Protein<br>Nucleotide<br>Ligand	 64.10<br>109.24<br>45.36	 43.13<br>84.86<br>41.18	 208.29<br>---<br>314.91	 144.11<br>---<br>113.93
R.m.s. deviations<br>Bond lengths (Å)<br>Bond angles (°)	 0.005<br>1.004	 0.007<br>1.048	 0.010<br>1.212	 0.004<br>0.910
Validation<br>MolProbity score<br>Clash score<br>Rotamer outliers (%)	 2.3<br>21.57<br>0.90	 2.33<br>20.99<br>1.22	 2.34<br>21.88<br>0.52	 2.18<br>16.50<br>1.16
Ramachandran plot<br>Favored (%)<br>Allowed (%)<br>Disallowed (%)	 92.50<br>7.02<br>0.48	 91.60<br>7.93<br>0.47	 91.52<br>8.07<br>0.41	 92.58<br>6.99<br>0.43
Model vs. Data<br>CC (mask)<br>CC (box)<br>CC (peak)<br>CC (volume)<br>Mean CC for ligands	 0.69<br>0.71<br>0.59<br>0.69<br>0.68	 0.75<br>0.71<br>0.61<br>0.72<br>0.71	 0.80<br>0.90<br>0.77<br>0.80<br>0.81	 0.79<br>0.81<br>0.71<br>0.79<br>0.85
